# Use of incretin-based drugs and risk of cholangiocarcinoma: Scandinavian cohort study

**DOI:** 10.1007/s00125-021-05508-1

**Published:** 2021-07-13

**Authors:** Peter Ueda, Viktor Wintzell, Mads Melbye, Björn Eliasson, Ann-Marie Svensson, Stefan Franzén, Soffia Gudbjörnsdottir, Kristian Hveem, Christian Jonasson, Henrik Svanström, Björn Pasternak

**Affiliations:** 1 Clinical Epidemiology Division, Department of Medicine, Solna, Karolinska Institutet, Stockholm, Sweden; 2 Department of Epidemiology Research, Statens Serum Institut, Copenhagen, Denmark; 3 Department of Clinical Medicine, University of Copenhagen, Copenhagen, Denmark; 4 Department of Medicine, Stanford University School of Medicine, Stanford, CA USA; 5 Department of Molecular and Clinical Medicine, Institute of Medicine, University of Gothenburg, Gothenburg, Sweden; 6 The Swedish National Diabetes Register, Västra Götalandsregionen, Gothenburg, Sweden; 7 Health Metrics, Department of Public Health and Community Medicine, Sahlgrenska Academy, University of Gothenburg, Gothenburg, Sweden; 8 K.G. Jebsen Center for Genetic Epidemiology, Department of Public Health and Nursing, Faculty of Medicine and Health Science, NTNU—Norwegian University of Science and Technology, Trondheim, Norway; 9 HUNT Research Center, Faculty of Medicine, NTNU—Norwegian University of Science and Technology, Levanger, Norway; 10 Division of Health Data and Digitalization, The Norwegian Institute of Public Health, Oslo, Norway

**Keywords:** Cholangiocarcinoma, Dipeptidyl peptidase 4 inhibitors, DPP4 inhibitors, Drug safety, GLP-1-receptor-agonists, Glucagon-like peptide-1-receptor agonists, Type 2 diabetes

## Abstract

**Aims/hypothesis:**

Concerns have been raised regarding a potential association of use of the incretin-based drugs dipeptidyl peptidase 4 (DPP4) inhibitors and glucagon-like peptide-1 (GLP-1)-receptor agonists with risk of cholangiocarcinoma. We examined this association in nationwide data from three countries.

**Methods:**

We used data from nationwide registers in Sweden, Denmark and Norway, 2007–2018, to conduct two cohort studies, one for DPP4 inhibitors and one for GLP-1-receptor agonists, to investigate the risk of incident cholangiocarcinoma compared with an active-comparator drug class (sulfonylureas). The cohorts included patients initiating treatment episodes with DPP4 inhibitors vs sulfonylureas, and GLP-1-receptor agonists vs sulfonylureas. We used Cox regression models, adjusted for potential confounders, to estimate hazard ratios from day 366 after treatment initiation to account for cancer latency.

**Results:**

The main analyses of DPP4 inhibitors included 1,414,144 person-years of follow-up from 222,577 patients receiving DPP4 inhibitors (median [IQR] follow-up time, 4.5 [2.6–7.0] years) and 123,908 patients receiving sulfonylureas (median [IQR] follow-up time, 5.1 [2.9–7.8] years) during which 350 cholangiocarcinoma events occurred. Use of DPP4 inhibitors, compared with sulfonylureas, was not associated with a statistically significant increase in risk of cholangiocarcinoma (incidence rate 26 vs 23 per 100,000 person-years; adjusted HR, 1.15 [95% CI 0.90, 1.46]; absolute rate difference 3 [95% CI -3, 10] events per 100,000 person-years). The main analyses of GLP-1-receptor agonists included 1,036,587 person-years of follow-up from 96,813 patients receiving GLP-1-receptor agonists (median [IQR] follow-up time, 4.4 [2.4–6.9] years) and 142,578 patients receiving sulfonylureas (median [IQR] follow-up time, 5.5 [3.2–8.1] years) during which 249 cholangiocarcinoma events occurred. Use of GLP-1-receptor agonists was not associated with a statistically significant increase in risk of cholangiocarcinoma (incidence rate 26 vs 23 per 100,000 person-years; adjusted HR, 1.25 [95% CI 0.89, 1.76]; absolute rate difference 3 [95% CI -5, 13] events per 100,000 patient-years).

**Conclusions/interpretation:**

In this analysis using nationwide data from three countries, use of DPP4 inhibitors and GLP-1-receptor agonists, compared with sulfonylureas, was not associated with a significantly increased risk of cholangiocarcinoma.

**Graphical abstract:**

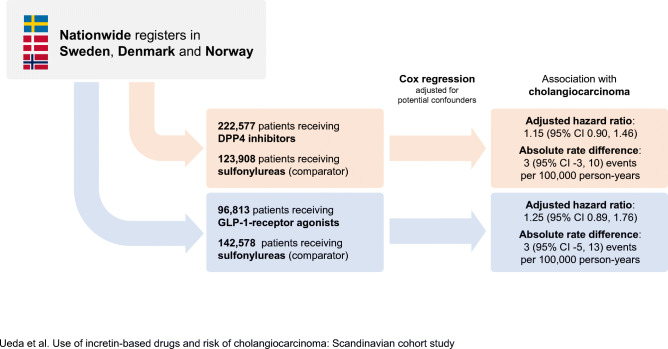

**Supplementary Information:**

The online version contains peer-reviewed but unedited supplementary material available at 10.1007/s00125-021-05508-1.

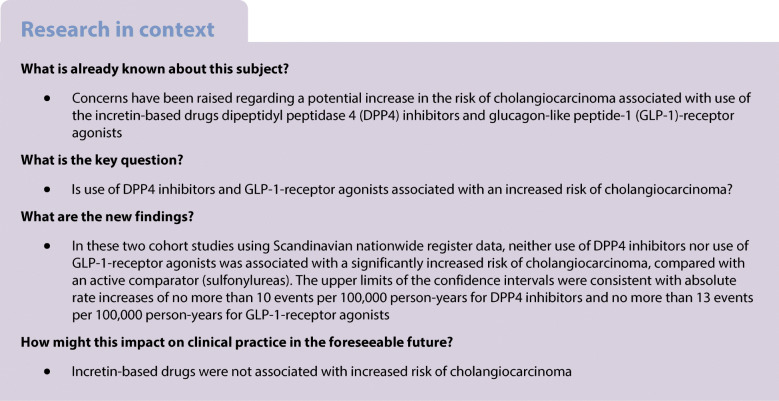



## Introduction

The incretin-based drug classes, dipeptidyl peptidase 4 (DPP4) inhibitors and glucagon-like peptide-1 (GLP-1)-receptor agonists, are commonly used for treatment of type 2 diabetes. European and US clinical guidelines now recommend GLP-1-receptor agonists for cardiovascular disease prevention among patients at high risk [1–4].

Recently, concerns have been raised regarding a potential association between incretin-based drugs and cholangiocarcinoma. These concerns were initially based on biological evidence indicating that the incretin system and the GLP-1-receptor might play a role in the development of cholangiocarcinoma [5–7], and further highlighted by an observational study using data from the UK Clinical Practice Research Datalink [8] showing that use of DPP4 inhibitors, compared with other second- or third-line glucose-lowering drugs, was associated with an increased risk of cholangiocarcinoma (HR 1.77 [95% CI 1.04, 3.01]). Analyses of GLP-1-receptor agonists were similarly suggestive of an increased risk although confidence intervals were wide, with only seven events occurring in the exposed group (HR 1.97 [95% CI 0.83, 4.66]). While the low number of events of cholangiocarcinoma in clinical trials precludes informative analyses [9–14], this safety signal is currently being monitored by the European Medicines Agency [15, 16].

To provide further data on this safety concern, we used nationwide registers in Sweden, Denmark and Norway to assess the association of use of DPP4 inhibitors and GLP-1-receptor agonists with risk of cholangiocarcinoma.

## Methods

### Data sources

Data sources are described in the electronic supplementary material (ESM) Methods. In brief, we used nationwide health and administrative registers in Sweden, Denmark and Norway, including population registers and Statistics Denmark/Statistics Sweden (vital status, demographics, socioeconomic variables), patient registers (comorbidities, outcomes), prescription registers (study drugs, co-medications) and the national cancer registers (outcome). The study was approved by the Regional Ethics Committee in Stockholm, Sweden; the Danish Data Protection Agency; and the Regional Committee for Medical and Health Research Ethics (REC Central), Norway. In Denmark, ethical approval is not required for register-based research. Informed consent was not required.

### Study population and exposure definitions

We conducted two separate cohort studies for the analyses of DPP4 inhibitors and GLP-1-receptor agonists, respectively. To reduce the risk of confounding by indication, severity of disease and unmeasured participant characteristics, we used an active-comparator design. The active comparator is ideally a drug that is used in the same clinical situation as the study drug while having no association with the outcome. We used sulfonylureas as the active comparator as clinical guidelines used during the study period recommended DPP4 inhibitors and GLP-1-receptor agonists as well as sulfonylureas as second- or third-line glucose-lowering therapies [17], and no concern regarding an increased risk of cholangiocarcinoma with use of sulfonylureas has been raised.

We based the analyses on treatment episodes. In each study cohort, we identified all treatment episodes of new use of the incretin-based drug of interest and of a sulfonylurea during the study period (2007 to the end of 2018 in Sweden and Denmark and 2010 to the end of 2018 in Norway). The date of filling the prescription constituted cohort entry. New use was defined as not having filled a prescription for the same drug at any time prior to cohort entry. The anatomic therapeutic chemical codes for the study drugs are shown in ESM Table 1. Patients who entered the cohort with a prescription for the incretin-based drug were considered as exposed to this drug until the end of follow-up regardless of whether they subsequently switched to or added a sulfonylurea drug. Patients who entered the cohort with a prescription for sulfonylureas and later switched to or added the incretin-based drug were considered as exposed to sulfonylureas from cohort entry to the date of filling the prescription for the incretin-based drug. Thereafter, this subsequent treatment episode with the incretin-based drug of interest was eligible for inclusion, with the date of filling the prescription constituting start of the treatment episode. Hence, a single participant could contribute with an episode of sulfonylurea use followed by an episode of incretin-based drug use, but not vice versa. In patients with two episodes, the episodes never overlapped in time and patients could not have more than one event, hence fulfilling assumptions of statistical independence (the occurrence of one outcome event could not affect the likelihood of another outcome event). We used this exposure categorisation to capture any exposure to the incretin-based drug and as use of sulfonylureas was not expected to be associated with the outcome. In a sensitivity analysis, we constructed cohorts in which each participant could only contribute with one treatment episode; these were defined based on the first treatment episode during the study period.

For each treatment episode, we applied exclusion criteria as defined in ESM Table 2. The exclusion criteria were selected to increase internal validity (rationales are shown in ESM Table 2) and included history of cholangiocarcinoma any time before cohort entry, healthcare visit for any cancer within the previous year, as well as diagnoses of human immunodeficiency virus, hepatitis B or C, cystic disease of the liver or choledochal duct, primary sclerosing cholangitis, cystic fibrosis, end-stage illness (e.g. cachexia and coma), drug misuse and severe pancreatic disorders (e.g. pancreatic enzyme substitution and major pancreatic surgery). We also excluded those without any specialist care contact or prescription drug use in the previous year (to exclude those with no encounters with the healthcare systems and those who recently immigrated and thus had incomplete covariate data).

### Outcome

The outcome was incident cholangiocarcinoma, identified from the national cancer registers in each country (ICD 10 codes: C22.1, C23 and C24). These registers collect information on all cancer cases, the majority of which are morphologically verified, and have high completeness and accuracy [18–20].

### Statistical analyses

We performed separate analyses for the cohorts comparing DPP4 inhibitor vs sulfonylureas use and GLP-1-receptor agonist vs sulfonylureas use. We pooled data from the three countries and used Cox regression models to estimate HRs for the risk of cholangiocarcinoma with use of each of the incretin-based drugs vs sulfonylureas. Patients were followed from the start of the treatment until the outcome event, death, emigration or end of the study period; as described above, treatment episodes with sulfonylureas were also censored at initiation of the incretin-based drug of interest in the respective cohort. The models used days since treatment initiation as the underlying time scale and were adjusted for country; age; sex; place of birth; cohabitation status; history of cardiovascular disease, diabetes complications, cancer (more than 1 year before cohort entry), gallbladder or pancreatic disorders, liver disease, inflammatory bowel disease and alcohol-related disorders; as well as diabetes medications and measures of healthcare utilisation, as measured at cohort entry (ESM Table 3). To account for cancer latency and to mitigate risk of bias due to early symptoms of the outcome that may affect treatment decisions, the HRs of the primary analysis were estimated from 1 year after treatment initiation. This corresponds to the use of a ‘lag period’ commonly employed in studies of cancer outcomes in pharmacoepidemiology [21]. Hence, those diagnosed with cholangiocarcinoma or meeting other censoring criteria during the lag period did not remain in the population that contributed to the estimation of the HR in the primary analysis. We estimated the absolute rate difference assuming a Poisson distribution. We presented the number and proportion of the outcome events by type of cholangiocarcinoma (extrahepatic, ICD 10 codes: C23, C24.0, C24.1; intrahepatic, ICD 10 code: C22.1; uncategorised, ICD 10 codes: C24.8, C24.9). We also performed separate analyses for each country to assess consistency across data sources. In additional analyses, we performed separate analyses by time since treatment initiation (1 to <3 years; 3 to <6 years; ≥6 years).

We performed sensitivity analyses. First, we estimated HRs without a lag period and with a 2 year lag period, respectively. Second, we performed analyses censoring follow-up at initiation of the other incretin-based drug (initiation of GLP-1-receptor agonists in analyses of DPP4 inhibitors and vice versa). Third, we additionally adjusted the analyses for calendar year at the start of the treatment episode as a categorical variable (2007–2009; 2010–2012; 2013–2015; 2016–2018). Fourth, we defined the study cohorts based on the first treatment episode during the study period, so that each participant could contribute to the cohort with no more than one treatment episode; here, cohorts were constructed excluding patients with previous use of any of the study drugs (the incretin-based drug or sulfonylureas) at cohort entry, with this exclusion being applied in addition to the exclusion criteria described in ESM Table 2. In this analysis patients were considered as exposed to the drug with which they entered the cohort until the end of follow-up. In essence, this analysis was a traditional active-comparator new-user design [22]. Fifth, we performed the analyses using sodium–glucose cotransporter 2 (SGLT2) inhibitors as the comparator: patients with no previous use of either of the study drugs and who initiated the incretin-based drug or an SGLT2 inhibitor from 1 April 2013 and onwards were included, with the date of filling the first prescription constituting cohort entry. Exclusion criteria (ESM Table 2) were applied. Patients were considered as exposed to the drug with which they entered the cohort until the end of follow-up. In addition to the variables shown in ESM Table 3, these analyses were adjusted for history of sulfonylureas use at cohort entry. Finally, we performed the analyses without applying the exclusion criteria related to risk factors of the outcome (ESM Table 2). All statistical analyses were done with SAS version 9.4 (SAS Institute, Cary, NC, USA).

## Results

### Study populations

**Fig. 1:**
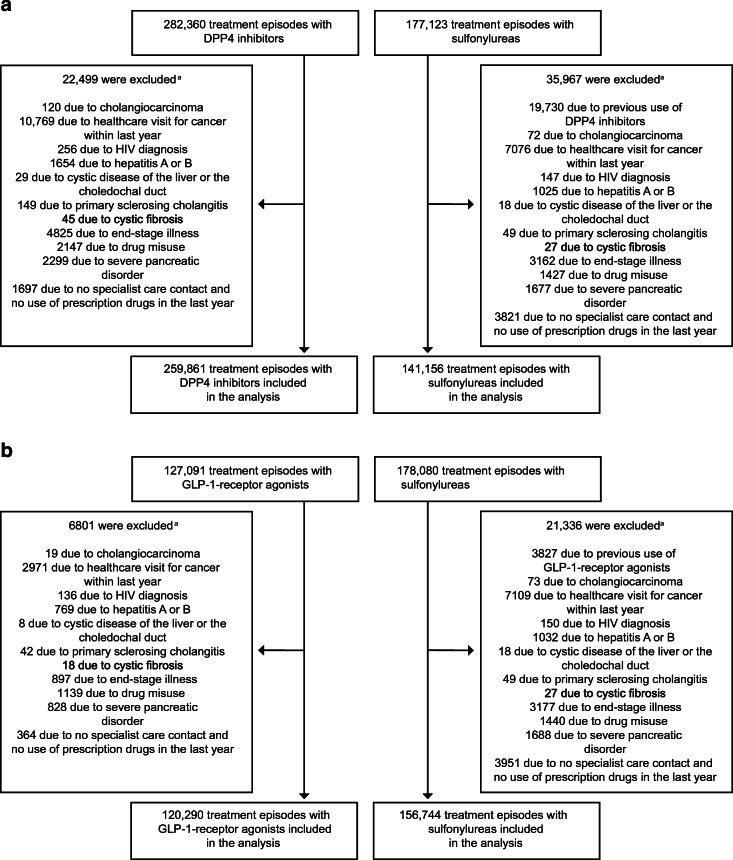
Flow chart of treatment episode inclusion in the study cohorts for analyses of (**a**) DPP4 inhibitors and (**b**) GLP-1-receptor agonists, respectively. ^a^Participants could be excluded for more than one reason

For the analyses of DPP4 inhibitors, 401,017 treatment episodes (*n* = 259,861 DPP4 inhibitor episodes; 141,156 sulfonylureas episodes) from 358,341 patients fulfilled the study eligibility criteria (Fig. 1; participant characteristics in Table 1). Among the episodes of DPP4 inhibitor use, mean (SD) age was 64.6 (11.9) and 40.5% were female. The corresponding numbers for episodes of sulfonylureas use were 64.6 (12.5) years and 43.1% female. Compared with treatment episodes with sulfonylureas, a larger proportion of the treatment episodes with DPP4 inhibitors were from patients with diabetes complications and with concurrent use of other glucose-lowering medications.

**Table 1:** Participant characteristics at cohort entry for the analyses of DPP4 inhibitors and GLP-1-receptor agonists, respectively

Characteristic	Cohort for analysis of DPP4 inhibitors		Cohort for analysis of GLP-1-receptor agonists	
	DPP4 inhibitors (n = 259,861)	Sulfonylureas (*n* = 141,156)	GLP-1-receptor agonists (n = 120,290)	Sulfonylureas (*n* = 156,744)
Country				
Sweden	111,812 (43.0)	78,368 (55.5)	53,071 (44.1)	84,196 (53.7)
Denmark	79,575 (30.6)	43,157 (30.6)	45,637 (37.9)	47,669 (30.4)
Norway	68,474 (26.4)	19,631 (13.9)	21,582 (17.9)	24,879 (15.9)
Age, mean (SD)	64.6 (11.9)	64.6 (12.5)	59.7 (10.8)	64.4 (12.4)
Female	105,369 (40.5)	60,808 (43.1)	52,315 (43.5)	67,057 (42.8)
Place of birth				
Scandinavia	217,825 (83.8)	116,738 (82.7)	104,609 (87.0)	129,135 (82.4)
Rest of Europe	16,840 (6.5)	9663 (6.8)	6739 (5.6)	10,930 (7.0)
Outside Europe	24,851 (9.6)	14,520 (10.3)	8820 (7.3)	16,411 (10.5)
Missing	345 (0.1)	235 (0.2)	122 (0.1)	268 (0.2)
Civil status				
Married/living with partner	147,639 (56.8)	77,328 (54.8)	68,727 (57.1)	86,172 (55.0)
Single	110,813 (42.6)	62,244 (44.1)	51,138 (42.5)	68,854 (43.9)
Missing	1409 (0.5)	1584 (1.1)	426 (0.4)	1718 (1.1)
Calendar year				
2007–2009	21,551 (8.3)	42,705 (30.3)	4625 (3.8)	43,443 (27.7)
2010–2012	64,544 (24.8)	46,393 (32.9)	32,317 (26.9)	50,183 (32.0)
2013–2015	75,697 (29.1)	32,092 (22.7)	29,927 (24.9)	37,443 (23.9)
2016–2018	98,069 (37.7)	19,966 (14.1)	53,421 (44.4)	25,675 (16.4)
Comorbidities				
Cardiovascular disease	84,648 (32.6)	43,224 (30.6)	37,538 (31.2)	47,529 (30.3)
Diabetic complications	91,907 (35.4)	37,985 (26.9)	52,178 (43.4)	42,610 (27.2)
Cancer (excl. non-melanoma skin cancer)^a^	13,092 (5.0)	6745 (4.8)	5100 (4.2)	7449 (4.8)
Gall bladder or pancreatic disorders	11,188 (4.3)	6735 (4.8)	5902 (4.9)	7380 (4.7)
Liver disease	3645 (1.4)	1880 (1.3)	2103 (1.7)	2084 (1.3)
Inflammatory bowel disease	5722 (2.2)	3030 (2.1)	2989 (2.5)	3352 (2.1)
Other alcohol-related disorders	3997 (1.5)	2436 (1.7)	1979 (1.6)	2621 (1.7)
Healthcare utilisation in last year				
Hospitalisation due to type 2 diabetes	5021 (1.9)	3361 (2.4)	2924 (2.4)	3598 (2.3)
Hospitalisation due to other causes	51,344 (19.8)	28,468 (20.2)	21,973 (18.3)	30,985 (19.8)
Outpatient contact due to type 2 diabetes	41,822 (16.1)	11,932 (8.5)	33,269 (27.7)	14,844 (9.5)
Outpatient contact due to other causes	139,964 (53.9)	71,815 (50.9)	71,868 (59.7)	79,705 (50.9)
Diabetes drugs in last 6 months				
None	26,663 (10.3)	40,538 (28.7)	9284 (7.7)	41,371 (26.4)
Metformin	205,113 (78.9)	97,129 (68.8)	90,921 (75.6)	109,892 (70.1)
Insulin	35,831 (13.8)	6075 (4.3)	46,347 (38.5)	6385 (4.1)
Thiazolidinediones	9185 (3.5)	2332 (1.7)	2351 (2.0)	2592 (1.7)
Other antidiabetics (glinides, acarbose)	6647 (2.6)	2400 (1.7)	2969 (2.5)	2707 (1.7)
GLP-1-receptor agonists	4623 (1.8)	1595 (1.1)	–	–
DPP4 inhibitors	–	–	12,158 (10.1)	5541 (3.5)

Numbers are shown as *n* (%) unless indicated otherwise

^a^Recorded more than 1 year prior to start of treatment episode

For the analyses of GLP-1-receptor agonists, 277,034 treatment episodes (*n* = 120,290 GLP-1-receptor agonist episodes; 156,744 sulfonylureas episodes) from 252,861 patients were included (Fig. 1; participant characteristics in Table 1). Among episodes of GLP-1-receptor agonist use, mean (SD) age was 59.7 (10.8) years and 43.5% were female. The corresponding numbers for episodes of sulfonylureas use were 64.4 (12.4) years and 42.8% female. Also in this study population, a larger proportion of treatment episodes with GLP-1-receptor agonists were from patients with diabetes complications and with concurrent use of other glucose-lowering medications.

### Primary analyses

**Fig. 2:**
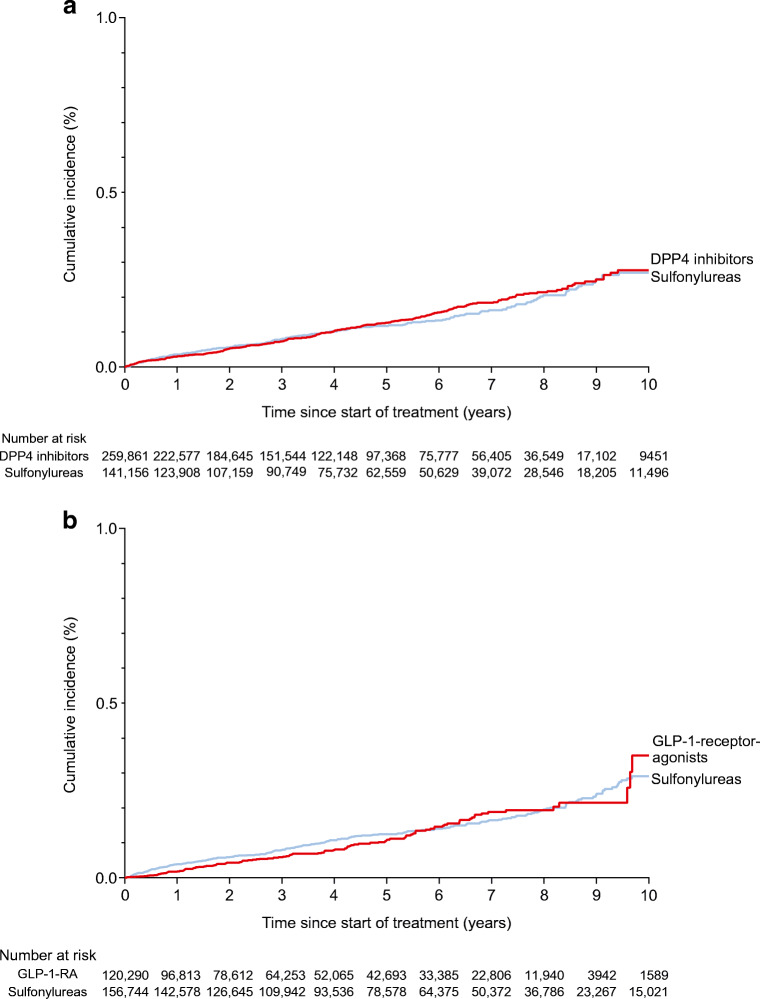
Cumulative incidence of cholangiocarcinoma in the analysis of (**a**) DPP4 inhibitors and (**b**) GLP-1-receptor agonists, respectively, vs sulfonylureas. Because of declining numbers of patients at risk and outcome events, cumulative incidence curves were truncated at 10 years (maximum follow-up in the study was 12 years). GLP-1-RA, GLP-1-receptor agonists

Table 2 shows the results of the primary analyses for DPP4 inhibitors and GLP-1-receptor agonists and Fig. 2 shows the cumulative incidence of cholangiocarcinoma. In the analyses of DPP4 inhibitors, 222,577 treatment episodes of DPP4 inhibitor use (median [IQR] follow-up time, 4.5 [2.6–7.0] years) and 123,908 treatment episodes of sulfonylurea use (median [IQR] follow-up time, 5.1 [2.9–7.8] years) remained at risk at 1 year after start of follow-up. During 1,414,144 person-years of follow-up, cholangiocarcinoma occurred among 222 patients with use of DPP4 inhibitors (incidence rate, 26 per 100,000 patient-years) and among 128 patients with use of sulfonylureas (incidence rate, 23 per 100,000 patient-years). The adjusted HR for use of DPP4 inhibitors vs sulfonylureas was 1.15 (95% CI 0.90, 1.46) and the absolute difference was 3 (95% CI -3, 10) events per 100,000 person-years.

**Table 2:** Association between use of DPP4 inhibitors and GLP-1-receptor agonists, respectively, and risk of cholangiocarcinoma in the primary analyses

Analysis	n	Events	Events per 100,000 person-years	Unadjusted HR (95% CI)	Adjusted^a^ HR (95% CI)	Adjusted^a^ absolute rate difference, events per 100,000 person-years
Analysis of DPP4 inhibitors						
DPP4 inhibitors	222,577	222	26	1.13 (0.91, 1.41)	1.15 (0.90, 1.46)	3 (−3, 10)
Sulfonylureas	123,908	128	23	[reference]	[reference]	[reference]
Analysis of GLP-1-receptor agonists						
GLP-1-receptor agonists	96,813	92	26	1.16 (0.89, 1.51)	1.25 (0.89, 1.76)	3 (−5, 13)
Sulfonylureas	142,578	157	23	[reference]	[reference]	[reference]

^a^Adjusted for country, age, sex, place of birth and cohabitation status; history of cardiovascular disease, diabetes complications, cancer (more than 1 year before cohort entry), gallbladder or pancreatic disorders, liver disease, inflammatory bowel disease and alcohol-related disorders; as well as diabetes medications and measures of healthcare utilisation

In the analyses of GLP-1-receptor agonists, 96,813 treatment episodes of GLP-1-receptor agonist use (median [IQR] follow-up time, 4.4 [2.4–6.9] years) and 142,578 treatment episodes of sulfonylurea use (median [IQR] follow-up time, 5.5 [3.2–8.1] years) remained at risk at 1 year after start of follow-up. During 1,036,587 person-years of follow-up, cholangiocarcinoma occurred among 92 patients with use of GLP-1-receptor agonists (incidence rate, 26 per 100,000 patient-years) and among 157 patients with use of sulfonylureas (incidence rate, 23 per 100,000 patient-years). The adjusted HR for use of GLP-1-receptor agonists vs sulfonylureas was 1.25 (95% CI 0.89, 1.76) and the absolute difference was 3 (95% CI -5, 13) events per 100,000 person-years. The adjusted HR was similar in all countries (ESM Table 4). The number and proportion of events by type of cholangiocarcinoma in the primary analyses are shown in ESM Table 5.

The additional analyses by time since treatment initiation are shown in ESM Table 6. None of the analyses showed statistically significant associations with cholangiocarcinoma. The point estimate increased for a time since treatment initiation of 3 to <6 years for DPP4 inhibitors but decreased again for ≥6 years. In analyses of GLP-1-receptor agonists, the point estimate decreased with longer time since treatment initiation.

### Sensitivity analyses

Results from the sensitivity analyses are shown in Tables 3 and 4. Varying the lag period, the adjusted HR vs sulfonylureas was 1.09 (95% CI 0.88, 1.33) for DPP4 inhibitors and 1.11 (95% CI 0.83, 1.49) for GLP-1-receptor agonists without a lag period and 1.11 (95% CI 0.85, 1.45) for DPP4 inhibitors and 1.06 (95% CI 0.72, 1.57) for GLP-1-receptor agonists with a 2 year lag period. When follow-up in the analysis of DPP4 inhibitors was censored at initiation of GLP-1-receptor agonists, the adjusted HR was 1.16 (95% CI 0.89, 1.50). The corresponding HR of GLP-1-receptor agonists when follow-up was censored at initiation of DPP4 inhibitors was 1.21 (95% CI 0.82, 1.80). In analyses additionally adjusted for calendar year, the adjusted HR was 1.14 (95% CI 0.89, 1.46) for DPP4 inhibitors and 1.25 (95% CI 0.88, 1.78) for GLP-1-receptor agonists. The analyses based on a traditional active-comparator new-user design using the first treatment episode during the study period included 131,298 new users of DPP4 inhibitors vs 133,233 new users of sulfonylureas and 53,302 new users of GLP-1-receptor agonists vs 146,294 new users of sulfonylureas who remained at risk at 1 year after start of follow-up (ESM Fig. 1; baseline characteristics in ESM Table 7). Hence, in these cohorts, the eligibility criterion requiring no use of the incretin-based drug of interest and the comparator prior to cohort entry led to a substantial exclusion of patients initiating an incretin-based drug because of a history of previous sulfonylureas use. In these analyses, the adjusted HR was 1.16 (95% CI 0.90, 1.49) for DPP4 inhibitors and 1.39 (95% CI 0.94, 2.07) for GLP-1-receptor agonists. The analyses using SGLT2 inhibitors as the comparator included 127,842 new users of DPP4 inhibitors (median [IQR] follow-up time, 3.5 [2.2–4.9] years) vs 38,667 new users of SGLT2 inhibitors (median [IQR] follow-up time, 2.5 [1.7–3.7] years) and 51,064 new users of GLP-1-receptor agonists (median [IQR] follow-up time, 3.2 [2.0–4.7] years) vs 57,736 new users of SGLT2 inhibitors (median [IQR] follow-up time, 2.5 [1.7–3.8] years) who remained at risk at 1 year after start of follow-up (ESM Fig. 2; baseline characteristics in ESM Table 8). In these analyses the adjusted HR was 0.95 (95% CI 0.50, 1.80) for DPP4 inhibitors and 0.96 (95% CI 0.52, 1.78) for GLP-1-receptor agonists. In the analyses in which the exclusion criteria related to risk factors of cholangiocarcinoma were not applied, the adjusted HR vs sulfonylureas was 1.15 (95% CI 0.91, 1.45) for DPP4 inhibitors and 1.19 (95% CI 0.85, 1.66) for GLP-1-receptor agonists.

**Table 3:** Sensitivity analyses of the association between use of DPP4 inhibitors and risk of cholangiocarcinoma

Analysis	Drug	n	Events	Events per 100,000 person-years	Unadjusted HR (95% CI)	Adjusted^a^ HR (95% CI)
No lag period	DPP4 inhibitors	259,861	296	27	1.04 (0.86, 1.26)	1.09 (0.88, 1.33)
	Sulfonylureas	141,156	177	26	[reference]	[reference]
2 year lag period	DPP4 inhibitors	184,645	176	27	1.13 (0.89, 1.44)	1.11 (0.85, 1.45)
	Sulfonylureas	107,159	105	24	[reference]	[reference]
Censor at use of GLP-1-receptor agonists	DPP4 inhibitors	209,240	184	26	1.18 (0.93, 1.49)	1.16 (0.89, 1.50)
	Sulfonylureas	120,534	114	23	[reference]	[reference]
Adjust for calendar year	DPP4 inhibitors	222,577	222	26	1.13 (0.91, 1.41)	1.14 (0.89, 1.46)
	Sulfonylureas	123,908	128	23	[reference]	[reference]
First treatment episode with traditional active-comparator new-user design	DPP4 inhibitors	131,298	118	25	1.12 (0.88, 1.42)	1.16 (0.90, 1.49)
	Sulfonylureas	133,233	172	24	[reference]	[reference]
SGLT2 inhibitors as comparator	DPP4 inhibitors	127,842	66	24	1.05 (0.59, 1.86)	0.95 (0.50, 1.80)
	SGLT2 inhibitors	38,667	14	23	[reference]	[reference]
Not applying exclusion criteria related to risk factors of cholangiocarcinoma	DPP4 inhibitors	231,414	237	27	1.15 (0.93, 1.43)	1.15 (0.91, 1.45)
	Sulfonylureas	129,024	134	23	[reference]	[reference]

^a^Adjusted for country, age, sex, place of birth and cohabitation status; history of cardiovascular disease, diabetes complications, cancer (more than 1 year before cohort entry), gallbladder or pancreatic disorders, liver disease, inflammatory bowel disease and alcohol-related disorders; as well as diabetes medications and measures of healthcare utilisation. Analyses using SGLT2 inhibitors as the comparator were also adjusted for use of sulfonylureas. In analyses in which exclusion criteria related to risk factors of cholangiocarcinoma were not applied, healthcare visit for any cancer (except non-melanoma skin cancer) within the previous year was included in the variable for history of cancer; HIV, hepatitis B or C, congenital cystic disease of the liver or choledochal duct, primary sclerosing cholangitis and cystic fibrosis before cohort entry were adjusted for as a single variable due to the low number of risk factor-exposed cases

**Table 4:** Sensitivity analyses of the association between use of GLP-1-receptor agonists and risk of cholangiocarcinoma

Analysis	Drug	n	Events	Events per 100,000 person-years	Unadjusted HR (95% CI)	Adjusted^a^ HR (95% CI)
No lag period	GLP-1-receptor agonists	120,290	111	24	0.93 (0.74, 1.17)	1.11 (0.83, 1.49)
	Sulfonylureas	156,744	216	26	[reference]	[reference]
2 year lag period	GLP-1-receptor agonists	78,612	69	25	1.12 (0.83, 1.51)	1.06 (0.72, 1.57)
	Sulfonylureas	126,645	130	24	[reference]	[reference]
Censor at use of DPP4 inhibitors	GLP-1-receptor agonists	85,578	77	26	1.17 (0.87, 1.56)	1.21 (0.82, 1.80)
	Sulfonylureas	123,882	118	23	[reference]	[reference]
Adjust for calendar year	GLP-1-receptor agonists	96,813	92	26	1.16 (0.89, 1.51)	1.25 (0.88, 1.78)
	Sulfonylureas	142,578	157	23	[reference]	[reference]
First treatment episode with traditional active-comparator new-user design	GLP-1-receptor agonists	53,302	50	27	1.21 (0.88, 1.66)	1.39 (0.94, 2.07)
	Sulfonylureas	146,294	180	24	[reference]	[reference]
SGLT2 inhibitors as comparator	GLP-1-receptor agonists	51,064	24	23	0.86 (0.48, 1.54)	0.96 (0.52, 1.78)
	SGLT2 inhibitors	57,736	23	25	[reference]	[reference]
Not applying exclusion criteria related to risk factors of cholangiocarcinoma	GLP-1-receptor agonists	99,449	94	26	1.14 (0.88, 1.47)	1.19 (0.85, 1.66)
	SGLT2 inhibitors	148,454	165	24	[reference]	[reference]

^a^Adjusted for country, age, sex, place of birth and cohabitation status; history of cardiovascular disease, diabetes complications, cancer (more than 1 year before cohort entry), gallbladder or pancreatic disorders, liver disease, inflammatory bowel disease and alcohol-related disorders; as well as diabetes medications and measures of healthcare utilisation. Analyses using SGLT2 inhibitors as the comparator were also adjusted for use of sulfonylureas. In analyses in which exclusion criteria related to risk factors of cholangiocarcinoma were not applied, healthcare visit for any cancer (except non-melanoma skin cancer) within the previous year was included in the variable for history of cancer; HIV, hepatitis B or C, congenital cystic disease of the liver or choledochal duct, primary sclerosing cholangitis and cystic fibrosis before cohort entry were adjusted for as a single variable due to the low number of risk factor-exposed cases

## Discussion

We used nationwide registers in three Scandinavian countries to conduct two cohort studies that assessed the risk of cholangiocarcinoma associated with use of DPP4 inhibitors and GLP-1-receptor agonists, respectively. In analyses using sulfonylureas as the active comparator, there was no statistically significant increased risk of cholangiocarcinoma associated with either drug class over a median follow-up of 4.5 years.

Data from clinical trials regarding the association between use of incretin-based drugs and cholangiocarcinoma are conflicting, with the limited number of events and follow-up time precluding conclusive analyses. In the Liraglutide Effect and Action in Diabetes: Evaluation of Cardiovascular Outcome Results (LEADER) trial of the GLP-1-receptor agonist liraglutide, more biliary cancers were observed among those receiving active treatment vs placebo (six vs two events), with all but one event in the liraglutide group being cholangiocarcinomas [11]. In the Saxagliptin Assessment of Vascular Outcomes Recorded in Patients with Diabetes Mellitus–Thrombolysis in Myocardial Infarction (SAVOR-TIMI) 53 trial, however, fewer cases of hepatobiliary cancers occurred among those receiving the DPP-4 inhibitor saxagliptin vs placebo (nine vs 12); specific data for biliary cancers have not been reported [10]. A recent meta-analysis [9] identified three clinical trials of DPP4 inhibitors in which at least one case of cholangiocarcinoma was reported [12–14]; three and four events occurred among patients receiving DPP4 inhibitors and placebo, respectively.

An observational study by Abrahami et al. [8] has prompted the European Medicines Agency to monitor cholangiocarcinoma events with incretin-based drugs. The study found that use of DPP4 inhibitors, compared with other second- or third-line glucose-lowering drugs, was associated with a 77% increase in the risk of cholangiocarcinoma (HR 1.77 [95% CI 1.04, 3.01]), although the analysis only included 27 DPP4 inhibitor-exposed events. Our analyses of DPP4 inhibitors included 222 exposed events and did not show any statistically significant association with cholangiocarcinoma (HR vs sulfonylureas 1.15 [95% CI 0.90, 1.46]). In addition to the larger number of events in our study which allowed for analysis with more statistical power and higher precision, we used a different study population and study design; the analyses also differed in the covariates used for adjustment. Moreover, the findings of the two studies should be interpreted in the context of the uncertainty of the estimates. In the analyses of GLP-1-receptor agonists, the study by Abrahami et al. included only seven exposed events. Thus, the confidence intervals for the HR vs use of other second- or third-line glucose-lowering drugs were wide, although the point estimate indicated that also these drugs might be associated with an increased risk (HR 1.97 [95% CI 0.84, 4.66]). In our analyses of GLP-1-receptor agonists, which included 92 exposed events, the HR vs use of sulfonylureas was 1.25 (95% CI 0.89, 1.76). In our sensitivity analyses using SGLT2 inhibitors as the comparator, the HR was 0.95 (95% CI 0.50, 1.80) for DPP4 inhibitors and 0.96 (95% CI 0.52, 1.78) for GLP-1-receptor agonists. Nonetheless, both our analyses and those performed by Abrahami et al. indicate that potential absolute risk increases associated with use of incretin-based drugs, if any, are likely small.

Strengths of our study include the inclusion of nationwide cohorts of patients seen in routine clinical practice in three countries and the use of national cancer registers in which the majority of cancer cases are morphologically verified [18–20]. Moreover, the median follow-up in the primary analyses was 4.5 years for DPP4 inhibitors with 25% of the patients (i.e. almost 65,000 patients) having more than 7.0 years of follow-up. The corresponding numbers for GLP-1-receptor agonists were 4.4 and 6.9 years. While there was no indication of an increased risk even after 6 years or more since treatment initiation in our additional analyses (HR for DPP4 inhibitors 0.92 [95% CI 0.58, 1.47]; HR for GLP-1-receptor agonists 1.11 [95% CI 0.54, 2.25]), incretin-based drugs can be used as lifelong treatments and studies with longer follow-up time would be needed when such data are available. Moreover, to mitigate the risk of confounding by indication, severity of disease and unmeasured participant characteristics, we used an active-comparator design assessing risk of cholangiocarcinoma with sulfonylureas in the primary analyses. An additional strength was the use of an alternative comparator, SGLT2 inhibitors, in sensitivity analyses; like the incretin-based drugs, these drugs were introduced in clinical practice more recently than sulfonylureas. Our study has limitations. The exposure definition was based on filled prescriptions. Low adherence may have biased the findings towards the null. Moreover, a study has suggested that biliary cancers might be underreported in the Swedish cancer register [23]. Potential underreporting is unlikely to differ between users of incretin-based drugs and sulfonylureas and, in our study, the incidence of cholangiocarcinoma was similar or higher in Sweden compared with in Denmark and Norway. Finally, due to the observational nature of this study, residual and unmeasured confounding cannot be ruled out.

In conclusion, in this study using nationwide data from three Scandinavian countries and sulfonylureas as the comparator, neither use of DPP4 inhibitors nor use of GLP-1-receptor agonists was associated with a significantly increased risk of cholangiocarcinoma.

## Supplementary Information

ESM (PDF 638 kb)

## Data Availability

The datasets analysed during the current study are not publicly available because they are register-based and contain information about individual patients.

## Abbreviations

DPP4: Dipeptidyl peptidase 4
GLP-1: Glucagon-like peptide-1
SGLT2: Sodium–glucose cotransporter 2
