# Supplementary material for: Use of incretin-based drugs and risk of cholangiocarcinoma: Scandinavian cohort study
Source: Diabetologia. 2021 Jul 13;64(10):2204–14. doi: 10.1007/s00125-021-05508-1 (PMC8423638; doi:10.1007/s00125-021-05508-1)
Supplement: Supplementary file 1 — (PDF 638 kb) [file 125_2021_5508_MOESM1_ESM.pdf]

Electronic Supplementary Material (ESM)

Ueda P et al. Use of Incretin-based Drugs and Risk of Cholangiocarcinoma: Scandinavian Cohort Study

ESM Methods

Data sources

We used nationwide health and administrative registers in Sweden, Denmark and Norway. From the national prescription registers in each of the three countries, we obtained data on filled prescriptions.[1–4] These registers include information regarding all drug prescriptions filled (the anatomical therapeutic chemical [ATC] code of the dispensed drug and information about the amount of drug dispensed, and the date when the prescription was filled) at all pharmacies in the countries since July, 2005, in Sweden, 1995 in Denmark, and 2004 in Norway. From the national patient registers information on outpatient and emergency department visits and inpatient admissions to all public hospitals and private specialist care were obtained.[5–7] This information, which is registered as physician-assigned procedure codes, and diagnoses according to the International Classification of Diseases, tenth revision (ICD10), was used to obtain data about history of disease for each patient. The population registers of each country provided information on age, sex, country of birth, migration status, civil status (Norway) and vital status of the patients.[8–10] From Statistics Denmark and Statistics Sweden[8] we obtained data on patients' and civil status. The national cancer registers provided data on diagnoses of cholangiocarcinoma. Reporting to these is mandatory for every cancer diagnosed, with the majority of cases (89%-99%) being morphologically verified.[11–13]

ESM Table 1 ATC codes for DPP4 inhibitors, GLP1-receptor-agonists and sulfonylureas.

**Drug class**	**ATC-codes**
DPP4 inhibitors	A10BH, A10BD07, A10BD08, A10BD09, A10BD10, A10BD11, A10BD13, A10BD19, A10BD21, A10BD22, A10BD24, A10BD25
GLP1-receptor-agonists	A10BJ, A10AE54, A10AE56
Sulfonylureas	A10BB, A10BD01, A10BD02, A10BD04, A10BD06

ESM Table 2 ICD10 and procedure codes for exclusion criteria.

	Category	Codes (ICD-10, NCSP, or ATC)
1.	Cholangiocarcinoma any time before cohort entry	C22.1, C23, C24
2.	Healthcare visit for any cancer (except non-melanoma skin cancer) within previous year	C00-C43, C45-C97
3.	HIV before cohort entry[a]	B20-B24
4.	Hepatitis B or C before cohort entry[a]	B18.0, B18.1, B18.2
5.	Congenital cystic disease of the liver or choledochal duct[a]	Q44.4,[b] Q44.6[b]
6.	Primary sclerosing cholangitis before cohort entry[a]	K83.0A (K83.0 in Norway) [c]
7.	Cystic fibrosis before cohort entry[a]	E84
8.	Endstage illness (severe malnutrition, cachexia, dementia, coma) before cohort entry[a]	ICD-10: E40-E43, F00-F03, G30, R40.2[b], R64; ATC: N06D
9.	Drug misuse within last year before cohort entry	ICD-10: F11-F16, F18, F19, R78.1-R78.5, T40; [b] ATC: N07BB, N07BC
10.	Major pancreatic disease (chronic pancreatitis [defined by pancreatic enzyme substitution prescription within last year or diagnosis at any time before index date], pancreatic cancer, major pancreatic surgery at any time before cohort entry)[a]	ICD-10: C25, K86.0, K86.1; NCSP: JLC, JLE; ATC: A09AA02
11.	No hospital contact or prescription drug in last year prior to the cohort entry	n.a.

[a] 10-year look-back for Sweden and Denmark and 5-year look-back for Norway.
[b] Not available in Norwegian dataset.
[c] In Norway, the 4-level code (cholangitis) was used because 5-level data for this ICD10-code was not available.

Abbreviations: ICD, International Classification of Diseases; NCSP, NOMESCO Classification of Surgical Procedures; ATC, Anatomical Therapeutic Chemical

Rationale for exclusion criteria

Exclusion criteria constituted history of the outcome (1), relatively rare risk factors for the outcome or factors associated with the likelihood of detecting the outcome for which information regarding severity was not available (2)-(7), factors associated with the likelihood of receiving a diagnosis of the outcome or health seeking behavior (8)-(9) or potential use of the study drugs with an indication other than type 2 diabetes (10). Patients with no hospital contact or prescription drug in the last year (11) may have recently immigrated or have incomplete information regarding medical history, previous exposure to the study drugs and other information that we use in the analyses.

ESM Table 3 Definitions of variables adjusted for in the multivariable Cox regression models.

**Sociodemographic characteristics**	**ICD 10 codes/procedure codes/categories**
Age group	Cubic splines
Sex	Women/men
Place of birth	Scandinavia; Rest of Europe; Outside Europe, Missing
Living with partner	Yes/no
Country	Sweden; Denmark; Norway
**Medical history**[a]	
Cardiovascular disease	ICD-10: I110, I11, I20, I21, I22, I24, I25, I130, I132, I34-I37, I42, I43, I44-I47, I48, I49, I50, I60-I64, I65-I69, I70, I72, I73, I74, I77, J81, G45 (excl G454), G46, K550, K551, E115, E145, E135<br><br>Procedure codes: NFQ ,NGQ, NHQ
Other diabetes complications (renal complications, eye complications, neuropathy, hypoglycemia, metabolic acidosis, unspecified complications, foot complications)	ICD-10: E112, E142, I120, I131, I132, N00-08, N10-N16, N17, N18, N19, N20-N23, N25-N29, E110, E111, E113, E114, E116, E117, E118, E130, E131, E133, E134, E136, E137, E138, E140, E141, E143, E144, E146, E147, E148, E160, E161, E162, G990, G590, G632, H280[a], H358, H360, M142, M146, M908, L984<br><br>Procedure codes: CKC10, CKC12, CKC15, CKD65
Cancer (excl non-melanoma skin cancer)	ICD-10: C00-C43, C45-C97
Gall bladder or pancreatic disorders	K80-K87 (except K83.0A[c]), Q44[b] (except Q44.4 and Q44.6)
Liver disease	K70-K77, E83.1[b]
Inflammatory bowel disease	K50-K52
Other alcohol-related disorders	F10
**Diabetes drugs in the last 6 months**	
No diabetes drug	Not any A10
Metformin	A10BA02, A10BD07, A10BD08, A10BD10, A10BD11, A10BD13,<br>A10BD22, A10BD25, A10BD03, A10BD05, A10BD14, A10BD15,<br>A10BD16, A10BD20, A10BD23, A10BD02
Insulin	A10A, A10AE54, A10AE56
Thiazolidinediones	A10BG, A10BD03, A10BD04, A10BD05, A10BD06, A10BD09
Other antidiabetics (glinides, acarbose)	A10BF01, A10BD14, A10BX
GLP1-receptor-agonists[d]	A10BJ, A10AE54, A10AE56
DPP4 inhibitors[e]	A10BD07, A10BD08, A10BD09, A10BD10, A10BD11, A10BD13, A10BD19, A10BD21, A10BD22, A10BD24, A10BD25
**Health care utilization in previous year**	
Hospitalization due to type 2 diabetes	E11 (primary position)

Hospitalization due to other causes	Not E11 (primary position)
Outpatient contact due to type 2 diabetes	E11 (primary position)
Outpatient contact due to other causes	Not E11 (primary position)

[a] 10-year look-back for Sweden and Denmark and 5-year look-back for Norway.
[b] Not available in Norway.
[c] K83.0 in Norway.
[d] In analyses of DPP4 inhibitors.
[e] In analyses of GLP1-receptor-agonists.

ESM Table 4 Association between use of DPP4 inhibitors and GLP1-receptor-agonists, respectively, and risk of cholangiocarcinoma in each country.

		N	Events	Events per 100000 person-years	Unadjusted hazard ratio (95% CI)	Adjusted[a] hazard ratio (95% CI)
**Analysis of DPP4 inhibitors**						
Denmark	DPP4 inhibitors	70338	78	25	1.40 (0.94 to 2.10)	1.28 (0.82 to 2.00)
	Sulfonylureas	37797	34	18	[reference]	[reference]
Sweden	DPP4 inhibitors	91664	95	30	1.17 (0.87 to 1.58)	1.12 (0.81 to 1.54)
	Sulfonylureas	70009	82	26	[reference]	[reference]
Norway	DPP4 inhibitors	60575	49	21	0.99 (0.52 to 1.86)	1.01 (0.51 to 1.97)
	Sulfonylureas	16102	12	21	[reference]	[reference]
**Analysis of GLP1-receptor-agonists**						
Denmark	GLP1-receptor-agonists	38448	40	23	1.26 (0.81 to 1.96)	1.17 (0.67 to 2.04)
	Sulfonylureas	42791	42	19	[reference]	[reference]
Sweden	GLP1-receptor-agonists	40868	42	33	1.35 (0.93 to 1.96)	1.40 (0.87 to 2.24)
	Sulfonylureas	77457	96	26	[reference]	[reference]
Norway	GLP1-receptor-agonists	17497	10	17	0.78 (0.36 to 1.68)	0.79 (0.31 to 2.04)
	Sulfonylureas	22330	19	21	[reference]	[reference]

[a] Adjusted for age, sex, place of birth, cohabitation status, history of cardiovascular disease, diabetes complications, cancer (more than 1 year before cohort entry), gallbladder or pancreatic disorders, liver disease, inflammatory bowel disease and alcohol-related disorders, as well as diabetes medications and measures of healthcare utilization.

ESM Table 5 Number and proportion of cholangiocarcinoma by type for each study drug in the primary analyses. Numbers are shown in n (%)

	**Analyses of DPP4 inhibitors**		**Analyses of GLP1-receptor-agonists**	
	DPP4 inhibitors	Sulfonylureas	GLP1-receptor-agonists	Sulfonylureas
Extrahepatic	118 (53.2)	67 (52.3)	47 (51.1)	81 (51.6)
Intrahepatic	80 (36.0)	41 (32.0)	36 (39.1)	51 (32.5)
Uncategorized	24 (10.8)	20 (15.6)	9 (9.8)	25 (15.9)

ESM Table 6 Association between use of DPP4 inhibitors and GLP1-receptor-agonists, respectively, and risk of cholangiocarcinoma by time since treatment initiation.

		N	Events	Events per 100000 person-years	Unadjusted hazard ratio (95% CI)	Adjusted[a] hazard ratio (95% CI)
**Analysis of DPP4 inhibitors**						
1 to <3 years	DPP4 inhibitors	222577	80	22	0.99 (0.69 to 1.41)	1.03 (0.69 to 1.52)
	Sulfonylureas	123908	47	22	[reference]	[reference]
3 to <6 years	DPP4 inhibitors	151530	92	28	1.52 (1.04 to 2.21)	1.47 (0.97 to 2.22)
	Sulfonylureas	90719	38	18	[reference]	[reference]
≥6 years	DPP4 inhibitors	75765	50	32	0.94 (0.62 to 1.41)	0.92 (0.58 to 1.47)
	Sulfonylureas	50608	43	34	[reference]	[reference]
**Analysis of GLP1-receptor-agonists**						
1 to <3 years	GLP1-receptor-agonists	96813	34	21	1.04 (0.68 to 1.60)	1.40 (0.81 to 2.43)
	Sulfonylureas	142578	52	21	[reference]	[reference]
3 to <6 years	GLP1-receptor-agonists	64249	40	28	1.34 (0.89 to 2.01)	1.18 (0.69 to 2.03)
	Sulfonylureas	109922	54	21	[reference]	[reference]
≥6 years	GLP1-receptor-agonists	33374	18	32	1.08 (0.63 to 1.87)	1.11 (0.54 to 2.25)
	Sulfonylureas	64358	51	31	[reference]	[reference]

ESM Table 7 Patient characteristics of new users of DPP4 inhibitors and sulfonylureas and GLP1-receptor-agonists and sulfonylureas, respectively, for sensitivity analyses using a traditional active-comparator new-user design based on the first treatment episode during the study period.

	**Cohort for analysis of DPP4 inhibitors**		**Cohort for analysis of GLP1-receptor-agonists**	
	**DPP4 inhibitors** (N=157221)	**Sulfonylureas** (N=142057)	**GLP1-receptor-agonists** (N=68647)	**Sulfonylureas** (N=156890)
Country				
Sweden	73987 (47.1)	78716 (55.4)	34658 (50.5)	84250 (53.7)
Denmark	45385 (28.9)	43359 (30.5)	24289 (35.4)	47714 (30.4)
Norway	37849 (24.1)	19982 (14.1)	9700 (14.1)	24926 (15.9)
Age, mean (SD)	63.5 (12.0)	64.6 (12.5)	58.3 (10.9)	64.4 (12.4)
Female	63008 (40.1)	61128 (43.0)	30887 (45.0)	67125 (42.8)
Place of birth				
Scandinavia	133109 (84.7)	117353 (82.6)	60343 (87.9)	129255 (82.4)
Rest of Europe	10068 (6.4)	9753 (6.9)	3590 (5.2)	10942 (7.0)
Outside Europe	13868 (8.8)	14714 (10.4)	4658 (6.8)	16425 (10.5)
Missing	176 (0.1)	237 (0.2)	56 (0.1)	268 (0.2)
Civil status				
Married/living with partner	88471 (56.3)	77781 (54.8)	38307 (55.8)	86250 (55.0)
Single	67751 (43.1)	62649 (44.1)	30043 (43.8)	68916 (43.9)
Missing	999 (0.6)	1627 (1.1)	297 (0.4)	1724 (1.1)
Calendar year				
2007-2009	10781 (6.9)	42758 (30.1)	2208 (3.2)	43457 (27.7)
2010-2012	31770 (20.2)	46614 (32.8)	16005 (23.3)	50230 (32.0)
2013-2015	46336 (29.5)	32431 (22.8)	16592 (24.2)	37495 (23.9)
2016-2018	68334 (43.5)	20254 (14.3)	33842 (49.3)	25708 (16.4)
Comorbidities				
Cardiovascular disease	49446 (31.4)	43488 (30.6)	20481 (29.8)	47565 (30.3)

Diabetic complications	49227 (31.3)	38230 (26.9)	27027 (39.4)	42668 (27.2)
Cancer (excl non-melanoma skin cancer)[a]	7840 (5.0)	6766 (4.8)	2829 (4.1)	7456 (4.8)
Gall bladder or pancreatic disorders	7016 (4.5)	6775 (4.8)	3605 (5.3)	7387 (4.7)
Liver disease	2343 (1.5)	1897 (1.3)	1270 (1.9)	2087 (1.3)
Inflammatory bowel disease	3448 (2.2)	3044 (2.1)	1765 (2.6)	3355 (2.1)
Other alcohol-related disorders	2514 (1.6)	2456 (1.7)	1263 (1.8)	2624 (1.7)
Health care utilization in last year				
Hospitalization due to type 2 diabetes	3261 (2.1)	3459 (2.4)	1811 (2.6)	3611 (2.3)
Hospitalization due to other causes	30676 (19.5)	28706 (20.2)	12545 (18.3)	31013 (19.8)
Outpatient contact due to type 2 diabetes	21578 (13.7)	12101 (8.5)	15851 (23.1)	14883 (9.5)
Outpatient contact due to other causes	84732 (53.9)	72290 (50.9)	41535 (60.5)	79802 (50.9)
Diabetes drugs in last 6 months				
None	22782 (14.5)	40877 (28.8)	8503 (12.4)	41396 (26.4)
Metformin	125198 (79.6)	97640 (68.7)	49550 (72.2)	109986 (70.1)
Insulin	20600 (13.1)	6161 (4.3)	26489 (38.6)	6433 (4.1)
Thiazolidinediones	3746 (2.4)	2357 (1.7)	971 (1.4)	2596 (1.7)
Other antidiabetics (glinides, acarbose)	4515 (2.9)	2431 (1.7)	1963 (2.9)	2712 (1.7)
GLP-1-receptor-agonists	2310 (1.5)	1610 (1.1)	-	-
DPP4 inhibitors	-	-	5303 (7.7)	5552 (3.5)

[a] Recorded more than 1 year prior to start of treatment episode.

ESM Table 8 Patient characteristics of new users of DPP4 inhibitors and SGLT2 inhibitors and GLP1-receptor-agonists and SGLT2 inhibitors, respectively, for sensitivity analyses.

	**Cohort for analysis of DPP4 inhibitors**		**Cohort for analysis of GLP1-receptor-agonists**	
--	**DPP4 inhibitors** (N=160488)	**SGLT2 inhibitors** (N=61679)	**GLP1-receptor-agonists** (N=68350)	**SGLT2 inhibitors** (N=89876)
Country				
Sweden	78269 (48.8)	29839 (48.4)	36421 (53.3)	37872 (42.1)
Denmark	42085 (26.2)	17788 (28.8)	19626 (28.7)	25004 (27.8)
Norway	40134 (25.0)	14052 (22.8)	12303 (18.0)	27000 (30.0)
Age, mean (SD)	65.7 (12.2)	61.6 (10.8)	60.1 (11.2)	62.5 (10.8)
Female	65209 (40.6)	22732 (36.9)	30122 (44.1)	32714 (36.4)
Place of birth				
Scandinavia	133152 (83.0)	51307 (83.2)	58622 (85.8)	72749 (80.9)
Rest of Europe	10628 (6.6)	4086 (6.6)	4021 (5.9)	6549 (7.3)
Outside Europe	16532 (10.3)	6234 (10.1)	5652 (8.3)	10485 (11.7)
Missing	176 (0.1)	52 (0.1)	55 (0.1)	93 (0.1)
Civil status				
Married/living with partner	88301 (55.0)	34447 (55.8)	37426 (54.8)	51464 (57.3)
Single	71292 (44.4)	26959 (43.7)	30675 (44.9)	38029 (42.3)
Missing	895 (0.6)	273 (0.4)	249 (0.4)	383 (0.4)
Calendar year				
2013-2015	68973 (43.0)	12784 (20.7)	25765 (37.7)	19630 (21.8)
2016-2018	91515 (57.0)	48895 (79.3)	42585 (62.3)	70246 (78.2)
Comorbidities				
Cardiovascular disease	55430 (34.5)	21602 (35.0)	20936 (30.6)	29715 (33.1)
Diabetic complications	57739 (36.0)	23648 (38.3)	27940 (40.9)	32000 (35.6)
Cancer (excl non-melanoma skin cancer)[a]	9308 (5.8)	2753 (4.5)	3173 (4.6)	4010 (4.5)

Gall bladder or pancreatic disorders	7268 (4.5)	2786 (4.5)	3419 (5.0)	3648 (4.1)
Liver disease	2435 (1.5)	993 (1.6)	1264 (1.9)	1346 (1.5)
Inflammatory bowel disease	3855 (2.4)	1488 (2.4)	1771 (2.6)	1960 (2.2)
Other alcohol-related disorders	2633 (1.6)	997 (1.6)	1234 (1.8)	1335 (1.5)
Health care utilization in last year				
Hospitalization due to type 2 diabetes	3313 (2.1)	910 (1.5)	1525 (2.2)	1123 (1.2)
Hospitalization not due to type 2 diabetes causes	34214 (21.3)	11156 (18.1)	12303 (18.0)	14961 (16.6)
Outpatient contact due to type 2 diabetes	24041 (15.0)	12145 (19.7)	16288 (23.8)	18081 (20.1)
Outpatient contact not due to type 2 diabetes causes	89708 (55.9)	34724 (56.3)	41043 (60.0)	48616 (54.1)
Diabetes drugs in last 6 months				
No diabetes drug in last 6 months	20142 (12.6)	5219 (8.5)	7393 (10.8)	6010 (6.7)
Metformin	123457 (76.9)	49249 (79.8)	49043 (71.8)	73498 (81.8)
Insulin	23489 (14.6)	18242 (29.6)	26540 (38.8)	17251 (19.2)
Thiazolidinediones	965 (0.6)	519 (0.8)	592 (0.9)	853 (0.9)
Other antidiabetics (glinides, acarbose)	3495 (2.2)	1346 (2.2)	1536 (2.2)	2048 (2.3)
Sulphonylureas	33317 (20.8)	10401 (16.9)	12825 (18.8)	20088 (22.4)
GLP-1-receptor-agonists, no. (%)	3040 (1.9)	10346 (16.8)	-	-
DPP4 inhibitors, no. (%)	-	-	6215 (9.1)	15366 (17.1)

[a] Recorded more than 1 year prior to start of treatment episode.

ESM Figure 1 Flow chart of patient inclusion in the study cohort for sensitivity analyses of DPP4 inhibitors and GLP1-receptor-agonists, respectively, using a traditional active-comparator new-user design based on the first treatment episode during the study period.

DPP4 inhibitors

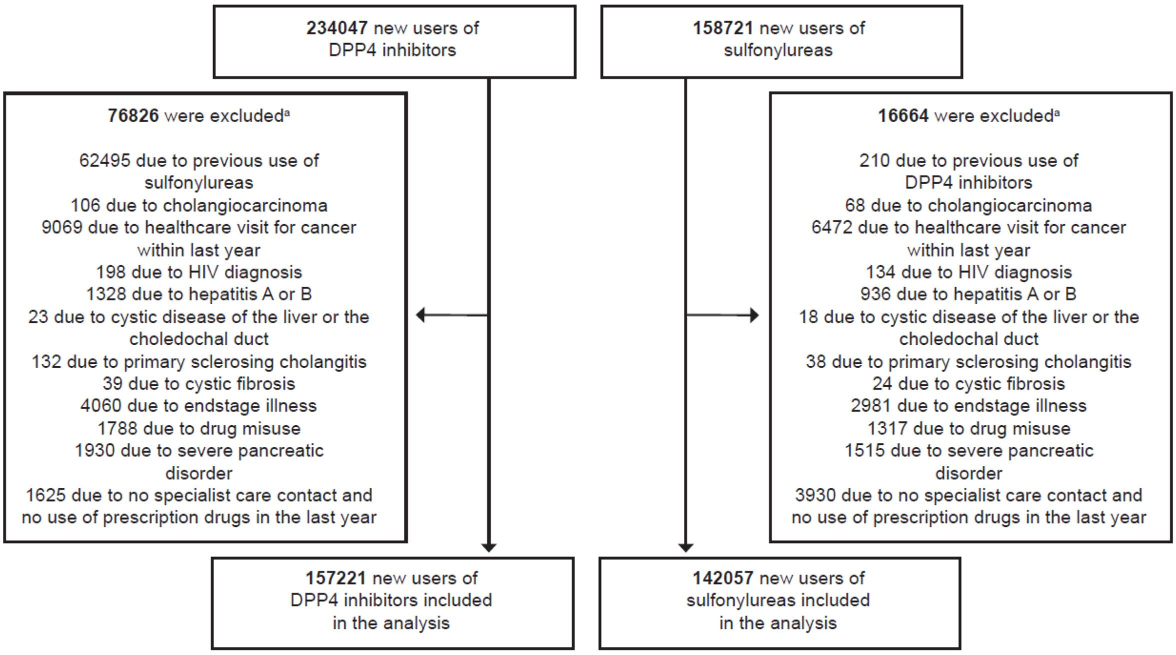

GLP1-receptor-agonists

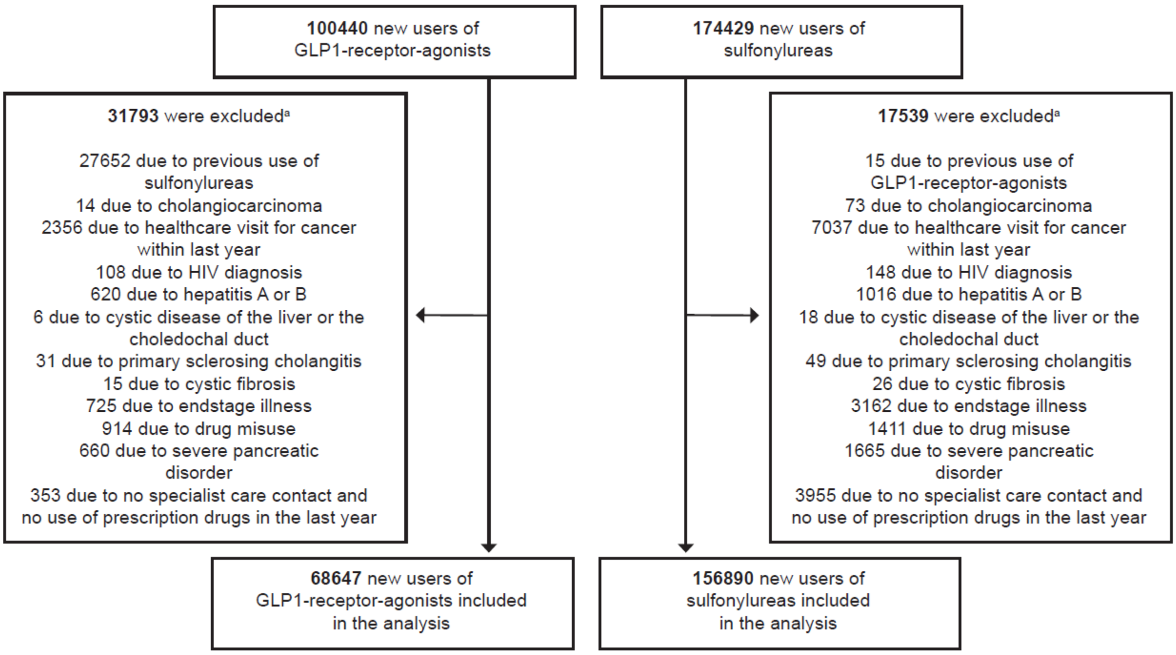

[a] One patient could be excluded due to more than one reason

ESM Figure 2 Flow chart of patient inclusion in the study cohort for sensitivity analyses of DPP4 inhibitors and GLP1-receptor-agonists, respectively, using SGLT2 inhibitors as the comparator.

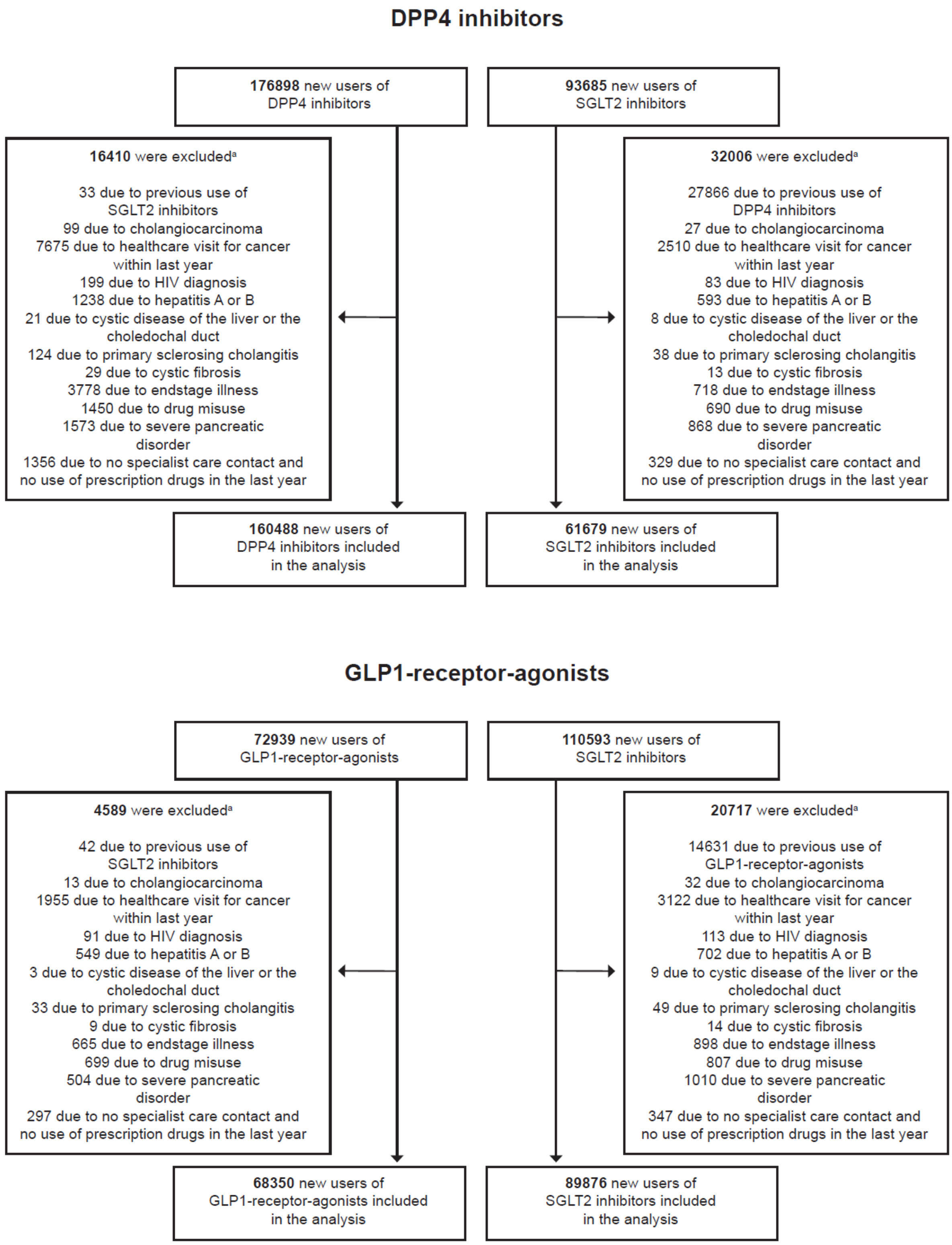

[a] One patient could be excluded due to more than one reason

References

1. Wettermark B, Hammar N, Fored M, et al. The new Swedish Prescribed Drug Register—Opportunities for pharmacoepidemiological research and experience from the first six months. *Pharmacoepidemiol Drug Saf*. 2007;16(7):726-735.
2. Pottegård A, Schmidt SAJ, Wallach-Kildemoes H, Sørensen HT, Hallas J, Schmidt M. Data Resource Profile: The Danish National Prescription Registry. *Int J Epidemiol*. 2016;46(3):dyw213.
3. Norwegian Institute of Public Health. Norwegian Prescription Database.
4. Furu K, Wettermark B, Andersen M, Martikainen JE, Almarsdottir AB, SÃ¸rensen HT. The Nordic Countries as a Cohort for Pharmacoepidemiological Research. *Basic Clin Pharmacol Toxicol*. 2010;106(2):86-94.
5. Ludvigsson JF, Andersson E, Ekbom A, et al. External review and validation of the Swedish national inpatient register. *BMC Public Health*. 2011;11(1):450.
6. Schmidt M, Schmidt SAJ, Sandegaard JL, Ehrenstein V, Pedersen L, Sørensen HT. The Danish National Patient Registry: a review of content, data quality, and research potential. *Clin Epidemiol*. 2015;7:449.
7. Norwegian Institute of Public Health. Overview of the national health registries. 2016.
8. Ludvigsson JF, Almqvist C, Bonamy A-KE, et al. Registers of the Swedish total population and their use in medical research. *Eur J Epidemiol*. 2016;31(2):125-136.
9. Schmidt M, Pedersen L, Sørensen HT. The Danish Civil Registration System as a tool in epidemiology. *Eur J Epidemiol*. 2014;29(8):541-549.
10. National Registry. The Norwegian Tax Administration.
11. Barlow L, Westergren K, Holmberg L, Tälback M. The completeness of the Swedish Cancer Register - A sample survey for year 1998. *Acta Oncol (Madr)*. 2009;48(1):27-33.
12. Gjerstorff ML. The Danish cancer registry. *Scand J Public Health*. 2011;39(7):42-45.
13. Larsen IK, Småstuen M, Johannesen TB, et al. Data quality at the Cancer Registry of Norway: An overview of comparability, completeness, validity and timeliness. *Eur J Cancer*. 2009;45(7):1218-1231.
